# Blackout in Spain: Urgent Analysis of Impact on Emergency Medical Services

**DOI:** 10.1017/S1049023X25101556

**Published:** 2025-12

**Authors:** Rafael Castro-Delgado, Youcef Azeli, Manuel Pardo Ríos, Joseph Cuthbertson, Ginés Martínez Bastida, Xavier Jiménez-Fábrega

**Affiliations:** 1. Department of Medicine, University of Oviedo, 33006 Oviedo, Spain; 2. Health Research Institute of the Principality of Asturias-ISPA (Research Group on Prehospital Care and Disasters, GIAPREDE), 33001, Oviedo, Spain; 3. Health Service of the Principality of Asturias (SAMU-Asturias), Oviedo, Spain; 4. RINVEMER-SEMES (Research Network on Prehospital Care-Spanish Society of Emergency Medicine), Madrid, Spain; 5. Sistema d’Emergències Mèdiques de Catalunya, Barcelona, Spain; 6. Emergency Department. Hospital Universitari Sant Joan, Reus, Spain; 7. Institut d’Investigació Sanitària Pere i Virgili (IISPV), Reus, Spain; 8. Universidad Católica de Murcia (UCAM), Murcia, Spain; 9. University of Notre Dame, Australia; 10. University of Barcelona, Barcelona, Spain

**Keywords:** blackout, emergency management, Emergency Medical Services

## Abstract

On April 28, 2025, a large-scale blackout affected mainland Spain and Portugal for over ten hours, severely impacting Emergency Medical Services (EMS). Although the cause remains uncertain and initially cyberattack was a concern, it has most probably been related to infrastructure failure. This event exposed critical vulnerabilities in EMS preparedness, as no region had a specific contingency plan for power outages.

The blackout led to wide-spread disruption, including traffic signal failures that caused accidents and delayed emergency response, and the collapse of communication networks that affected 1-1-2 emergency calls. Fuel shortages also emerged as gas stations became non-operational. Patients using home medical devices faced life-threatening situations, with at least one death reported due to a ventilator failure. The reliance on technology proved to be a major weakness, as many EMS systems lacked backup communication tools like satellite phones or analog radios, and many hospitals and ambulance bases were not prepared with stable generators and adequate fuel access.

Coordination between EMS, hospitals, and other emergency services was challenged by incompatible protocols and equipment. Despite these difficulties, EMS demonstrated adaptability by prioritizing urgent care and reallocating resources. The event exposed systemic fragilities and underscored the need for robust emergency planning, interagency drills, technological redundancy, and investment in resilient infrastructure. This incident serves as a global wake-up call, emphasizing that health systems must be prepared for increasing risks from climate change, cyber threats, and energy insecurity. Emergency preparedness should shift from being reactive to proactive, focusing on flexible systems, coordinated action, and workforce training to ensure continuity of health care during future blackouts.

## Introduction

On Monday April 28, 2025 at 12:33, a large-scale power outage on the Iberian Peninsula, which includes peninsular Spain and mainland Portugal, interrupted the electrical supply for ten or more hours.^1^ Potential causes continue to be analyzed. Initially, complex technical problems or cyberterrorism were investigated, with cyberterrorism being ruled out by Red Eléctrica Española (Alcobendas, Spain) on April 29, 2025.^2^ However, on June 17, 2025, an official government report established a technical reason as the main cause.^3^ At 22:55 on the day of the blackout, 55% of Spain’s national power supply had been restored.^4^

Spain, with a population of 48,619,695, is organized into 17 autonomous communities, each with its own public health service belonging to the National Health Service. Free health care is provided, covering primary, hospital, and emergency care. Each autonomous community has its own public Emergency Medical Services (EMS), including an Emergency Call Center (ECC). Before the 2025 Spanish blackout, blackouts had occurred in other areas leading to consequences for the population (Table 1), but this was the first time it had been studied at a national level with special emphasis on EMS.

**Table 1.** Previous Blackout Events with Consequences for the Population

Event	What Happened	Lessons Learned
Fukushima Daiichi, Japan (2011)	Following an earthquake and tsunami, a station blackout occurred and backup generators failed, leading to core meltdowns, hydrogen explosions, and release of radioactive material.	Highlights the need for robust emergency generators, redundancy, and plans for total grid failure. Training for simultaneous failures (electricity + communications + transport).
Winter Storm in Texas, USA (2021)	Severe cold weather caused the grid to collapse. Managed power outages were implemented, auxiliary infrastructures (water, heating) failed, and impacts were unequal across vulnerable populations.	Relevant for understanding secondary effects of a blackout: access to water, medical care, social inequality.
Sri Lanka Blackout (2020)	A technical transmission failure affected the entire country for hours. Basic services such as traffic, water, and communications were disrupted.	Recent example where a whole national electrical system went down, similar to Spain’s case.
South Australia Blackout (2016)	Severe storms damaged electrical infrastructure and triggered a state-wide blackout. Failures in forecasting extreme weather impacts.	Shows the importance of adapting infrastructure to extreme climate events and having contingency plans.

Emergency Medical Services plays a crucial role in global emergency crises and disasters like wildfires and the COVID-19 pandemic, as well as power outages.^5,6^ The United Nations Office for Disaster Risk Reduction (UNDRR; Geneva, Switzerland) Sendai Framework Terminology defines a blackout as a disruption to the electrical power supply to an end-user, causing a temporary or complete loss of power. It is classified as a technological hazard from infrastructure failure. Causal factors are varied and can include natural hazards or, more recently, cyberterrorism against power supplies.^7^ Consequently, it is essential to urgently share impacts that occurred for EMS in Spain during this blackout that inform the systemic risk profile of blackout incidents. Data were collected from members of the Prehospital Care and Disasters Research Network of the Spanish Society of Emergency Medicine (RINVEMER-SEMES; Madrid, Spain). This study has been approved by the Research Ethical Committee of the Universidad Católica de Murcia (Murcia, Spain; UCAM, ref. CE112309)

## Source

The primary sources of information were media and Spanish Government official information. Additionally, testimonials from members of the RINVEMER-SEMES were collected through internal reports.

## Observations

Observations have been organized in different components for a better understanding and approach.

### Risk Assessment and Planning

Large-scale power outages pose a significant threat to public safety and health care by disrupting services and operations. Despite the increased risk of such events, many EMS systems lack specific contingency plans. In Spain, no EMS had a contingency plan for a power outage or blackout.

### Logistical Challenges

The power outage in the Iberian Peninsula resulted in severe road traffic disruption due to non-functioning traffic signals, which caused numerous minor traffic accidents and delays in emergency vehicle movement. Individuals were also trapped in elevators, overwhelming fire and rescue teams. Fuel availability became critical as most gas stations could not operate without electricity. Some teams lacked access to reliable, independent communication tools, relying on mobile networks that quickly became saturated or failed. To support the surge in ambulance operational demand, non-urgent ambulance transport was temporarily ceased. Ambulances were monitored for their fuel supply, electromedicine batteries, and oxygen. Ambulances placed at hospitals could connect to the hospital’s electrical power provided by generators.

### Technological and Operational Resilience

In Spain, EMS activation typically relies on mobile networks or terrestrial trunked radio (TETRA) digital radio systems. While some services have access to very high frequency (VHF) radios, they are rarely used in day-to-day operations. During the blackout, personnel were often unfamiliar with the older systems. The 1-1-2 emergency number was compromised. Mobile network performance varied based on how long individual base stations could remain active without grid power. This autonomy depended on factors such as battery type and the availability of a generator at the site. Even though 1-1-2 calls can be routed through any available tower, there were still areas where calls would not connect. Other emergency numbers experienced even worse coverage failures. The fixed-line telephone network has also become less dependable during power failures as copper networks are replaced with fiber-optic infrastructure. No SMS-based mass alerts were issued during the event. Post-incident reports suggest that inconsistent network coverage would have hindered message delivery even if alerts had been sent. Communication failure during the blackout compromised the ability for the public to call EMS and for EMS to contact hospitals.

### Interagency Coordination

Spain’s response benefited from strong collaboration between EMS, police, firefighters, and utility companies. However, inconsistencies in communication protocols were evident. Even within the health care system, coordination was poor, and there were different approaches to the same challenges. For example, home patients on ventilators were managed by either hospital ventilator units or EMS, depending on the region.

### Health Impacts and Patient Care

**Figure 1.**
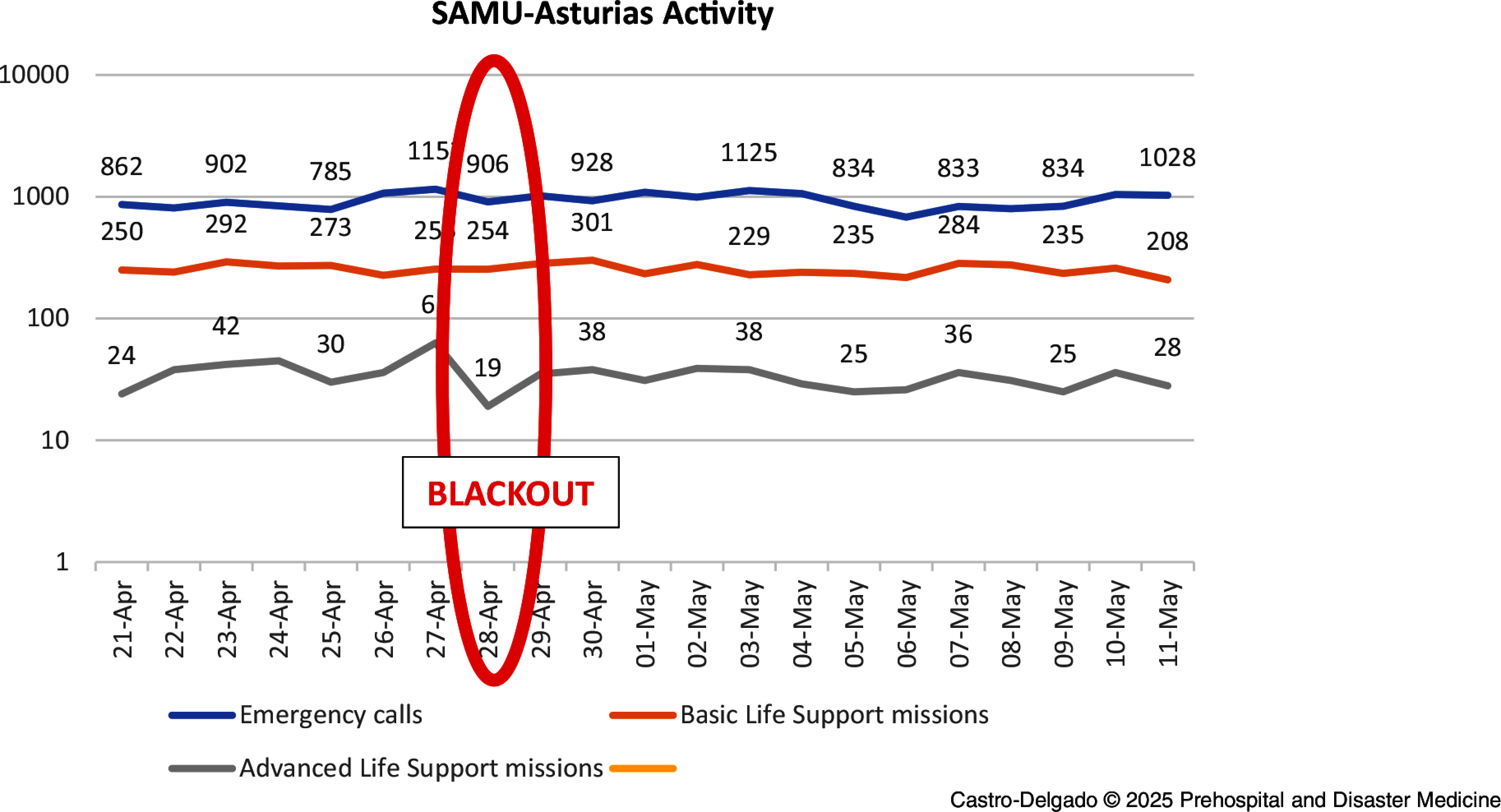
SAMU-Asturias Activity (for One Million Population). Abbreviation: SAMU, Servicio de Asistencia Médica Urgente [Emergency Medical Services].

There wasn’t a specific triaging of call procedure for blackouts, so normal protocols were used. Although global data in Spain are still to be analyzed, if data from Servicio de Asistencia Médica Urgente del Principado de Asturias (SAMU-Asturias; Oviedo, Spain) could be extrapolated (Figure 1), a slight decrease in total calls could be expected, possibly due to communication difficulties between the population and the emergency coordination center. It has also been identified that a decrease in emergency calls occurred at this time, a finding that requires further investigation. Patients relying on home-based medical devices, such as oxygen concentrators or ventilators, faced immediate risks. Many were evacuated or transferred to health care centers with backup power or self-transported themselves to a hospital. Home oxygen concentrators typically require a power connection and do not have a battery. Portable oxygen concentrators have a battery life of between two and eight hours. Patients dependent on home oxygen are also usually equipped with a supplemental oxygen cylinder, but its availability and size vary. Emergency departments experienced a rapid influx of these vulnerable individuals, which challenged their surge capacity. Diagnostic services and medical record access were maintained in many hospitals due to power generators. Some hospitals organized specific areas for these patients, and some ECCs organized a specific triage system for patients calling about a lack of energy to their home medical devices. At least one death was identified due to home ventilator failure during the blackout.^8^

### Staff Management

Call volume and care demand required the rapid mobilization of additional EMS and hospital personnel. However, communication infrastructure limitations hindered contact with off-duty staff. There was no dedicated emergency alert system independent of commercial mobile networks.

**Figure 2.**
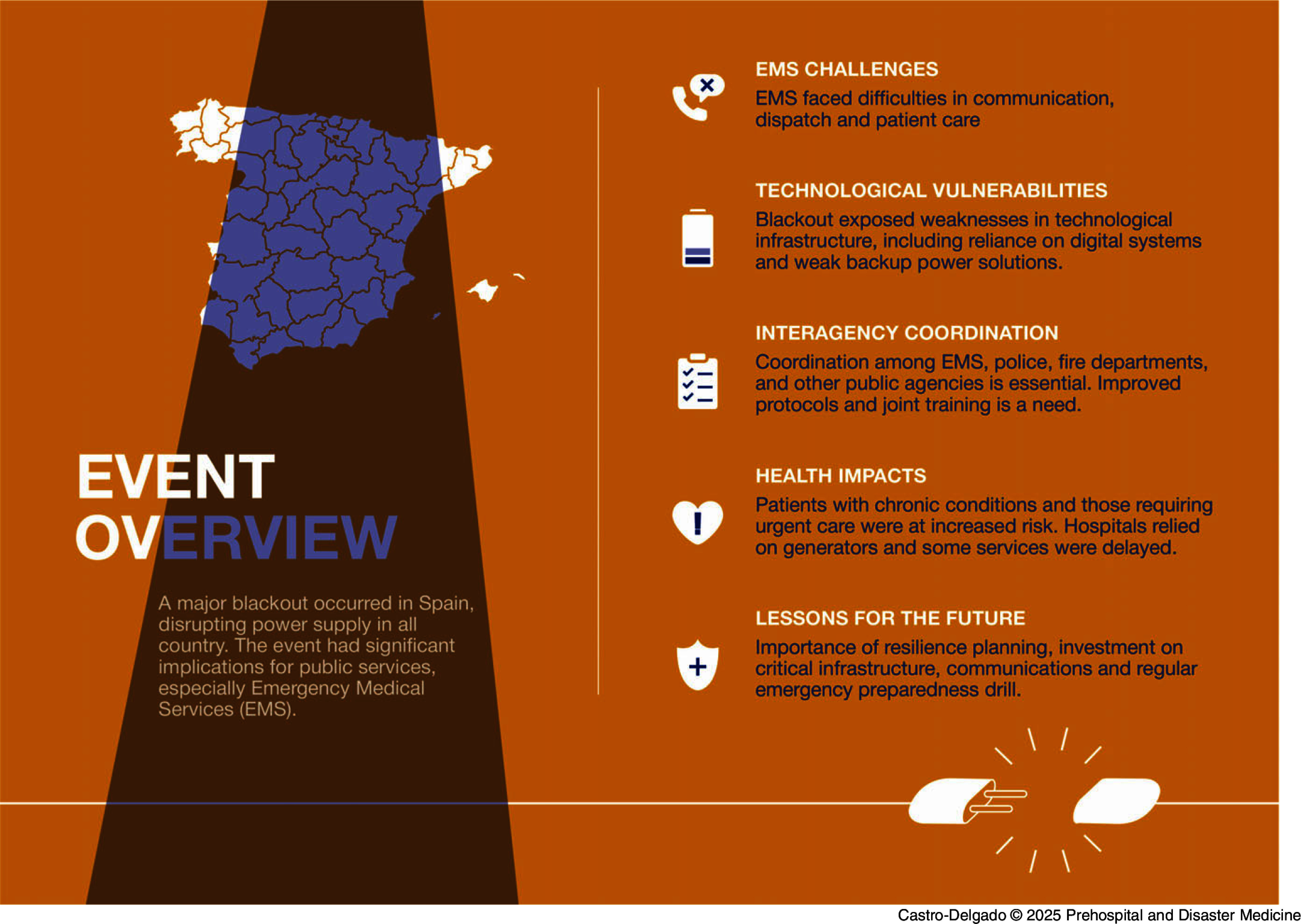
Main Findings Graphic. Abbreviation: EMS, Emergency Medical Services.

Table 2 describes identified problems caused by the blackout in the operation of the emergency medical systems. Figure 2 summarizes these findings in a visual format.

**Table 2.** Impact of the Blackout on the Emergency Medical Systems

Disruption in Communications
Partial failure of the 1-1-2 emergency telephone line
Partial failure of the population alert system using instant messaging
Alteration in communication between the ECC and ambulances
Alteration in communication between the ECC and the hospital network
Lack of awareness of available alternative media
Shortage of VHF stations
Lack of power supply in digital radio stations (TETRA)
**Failure in Ambulance Fuel Supply System**
No alternative plan for fuel supply
**Health Problems Associated with Lack of Electricity Supply**
Interruption of the operation of home oxygen concentrators
No access to oxygen cylinders or alternative energy generation methods
Failure of home mechanical ventilators
Concern and anxiety due to lack of knowledge about the battery life of ventilators
Poisoning from gases generated by diesel-powered electric generators (carbon monoxide)
**Disruption of Usual Patient Care**
Difficulty in assessing previous medical history due to failure to access the digital medical record, mainly in primary health care network
Up to five-times more alert calls to the ECC
Slowdown in hospital triage

Abbreviations: ECC, Emergency Call Center; VHF, very high frequency radio; TETRA, terrestrial trunked radio.

## Analysis: Lessons and Implications

Although the blackout in Spain was short-lived, its impact highlights the critical importance of emergency preparedness. Climate instability, energy insecurity, cyberattacks, and aging infrastructure increases the likelihood of these events. The experience of Spain’s EMS demonstrates that a lack of specific contingency plans for blackouts can lead to a reactive response, where actions are implemented as problems arise rather than mitigated or planned for prior through hazard and risk assessment processes.

A key weakness identified was the heavy reliance on stable power and communication systems, making EMS particularly vulnerable during wide-spread outages. This was evident in the disruption of many operational processes, such as dispatch systems and GPS navigation.

Specific actions to take into consideration for response to future blackouts related to EMS based on identified challenges faced during the Spanish blackout include:

1. Define specific actions plans to deal with blackouts;
2. Design resilient communication backup solutions (including satellite phones, analogue radios, and paper-based protocols);
3. Predefine EMS resources collection points; and
4. Design a power generator network for EMS resources provision, or keep records of vulnerable patients with home electromedicine devices, among others.

The incident also highlighted the essential role of coordinated action among emergency services. The strong collaboration that occurred was a key strength, but inconsistencies in communication protocols were a challenge. This underscores the importance of interagency drills that simulate wide-spread power failures to strengthen mutual understanding and efficiency.

The blackout serves as a compelling case study for health systems world-wide. The unique findings include the national-level impact analysis, the specific vulnerabilities exposed in Spain’s EMS, and the real-world consequences, such as the reported death from ventilator failure. The findings highlight the urgency of addressing the question “Are we ready to function effectively in the dark?” on a global scale.

Academic and research networks should prioritize studies into the impact of blackouts on health outcomes and system performance to develop evidence-based recommendations for future planning. Further analysis is proposed for the coming months, which includes contingency plans for incident command centers, identifying successful decisions made during the incident, and exploring the use of analogue devices as a backup for the EMS system.

Although the blackout in Spain was short-lived, its impact highlights why emergency preparedness is critical in the 21st Century. Climate instability, energy insecurity, cyberattacks, and aging infrastructure are making events like this increasingly likely. Emergency Medical Services across regions and countries must share knowledge and best practices for dealing with power outages.

This disaster report allows for making some reflections for an EMS call for action world-wide. Key effective actions—rapid triage using normal protocols, prioritization of urgent care, and interagency collaboration—proved life-saving at the time and should be scaled nation-wide. Immediate steps for EMS resilience include:

1. Develop blackout-specific contingency plans;
2. Invest in redundant communication systems (satellite phones, analogue radios, paper-based protocols);
3. Conduct regular interagency drills to standardize coordination across EMS, hospitals, and other health facilities;
4. Identify and support vulnerable patients relying on home medical devices, ensuring priority response; and
5. Expand technological and logistical redundancy (generators, fuel reserves, portable medical equipment), among others.

By scaling these strategies, EMS can transition from reactive crisis management to proactive preparedness, ensuring continuity of care during future blackouts.

## Conclusion

The April 2025 blackout in Spain was a wake-up call for EMS world-wide, revealing vulnerabilities for critical services. Spanish EMS professionals demonstrated commitment, adaptability, and resourcefulness. Based on the study’s findings, strengthening resilience in EMS requires a comprehensive, proactive approach that includes advance planning of operational capacity, human resources, and adaptable infrastructure. Inter-institutional coordination, articulated through regional command centers, is essential to anticipating the collapse of critical services and ensuring an integrated and efficient health response.

This study’s unique contribution is the national-level analysis of a blackout’s impact on EMS, highlighting the need for robust, blackout-specific contingency plans. The findings will improve care by providing evidence-based insights into systemic weaknesses and proactive strategies, such as technological redundancy and interagency drills, to ensure health care continuity during future blackouts.
